# Delayed Postconditioning Protects against Focal Ischemic Brain Injury in Rats

**DOI:** 10.1371/journal.pone.0003851

**Published:** 2008-12-10

**Authors:** Chuancheng Ren, Xuwen Gao, Gang Niu, Zhimin Yan, Xiaoyuan Chen, Heng Zhao

**Affiliations:** 1 Department of Neurosurgery, Stanford University, Stanford, California, United States of America; 2 Department of Radiology, Stanford University, Stanford, California, United States of America; 3 Department of Neurology, Shanghai No. 5 Hospital, Shanghai Medical School, Fudan University, Shanghai, China; Julius-Maximilians-Universität Würzburg, Germany

## Abstract

**Background:**

We and others have reported that rapid ischemic postconditioning, interrupting early reperfusion after stroke, reduces infarction in rats. However, its extremely short therapeutic time windows, from a few seconds to minutes after reperfusion, may hinder its clinical translation. Thus, in this study we explored if delayed postconditioning, which is conducted a few hours after reperfusion, offers protection against stroke.

**Methods and Results:**

Focal ischemia was generated by 30 min occlusion of bilateral common carotid artery (CCA) combined with permanent occlusion of middle cerebral artery (MCA); delayed postconditioning was performed by repetitive, brief occlusion and release of the bilateral CCAs, or of the ipsilateral CCA alone. As a result, delayed postconditioning performed at 3h and 6h after stroke robustly reduced infarct size, with the strongest protection achieved by delayed postconditioning with 6 cycles of 15 min occlusion/15 min release of the ipsilateral CCA executed from 6h. We found that this delayed postconditioning provided long-term protection for up to two months by reducing infarction and improving outcomes of the behavioral tests; it also attenuated reduction in 2-[^18^F]-fluoro-2-deoxy-D-glucose (FDG)-uptake therefore improving metabolism, and reduced edema and blood brain barrier leakage. Reperfusion in ischemic stroke patients is usually achieved by tissue plasminogen activator (tPA) application, however, t-PA's side effect may worsen ischemic injury. Thus, we tested whether delayed postconditioning counteracts the exacerbating effect of t-PA. The results showed that delayed postconditioning mitigated the worsening effect of t-PA on infarction.

**Conclusion:**

Delayed postconditioning reduced ischemic injury after focal ischemia, which opens a new research avenue for stroke therapy and its underlying protective mechanisms.

## Introduction

The current lack of clinical treatment for acute stroke necessitates the exploration of novel concepts that may eventually lead to clinical application. One of these concepts is ischemic postconditioning [1], which refers to an interference of a series of brief, repetitive occlusion and release of the cerebral blood vessels after reperfusion. We have demonstrated that rapid ischemic postconditioning performed immediately after reperfusion reduces infarction in focal cerebral ischemia [2], [3], [4], which has been confirmed by a number of other groups in global [5], [6] and focal ischemia [7], [8]. However, the extremely short therapeutic time windows may hinder its clinical translation; this specific limitation may prevent its application to those patients in whom reperfusion cannot be immediately and accurately identified.

On the other hand, it has been reported that delayed postconditioning conducted 2 d after transient global ischemia attenuates hippocampal injury in gerbils [9], [10]; here, delayed postconditioning was conducted by either mechanical occlusion or the neurotoxicant, 3-nitropropionic acid (3-NP). Whether delayed postconditioning attenuates brain injury after focal ischemia is not known. It is unlikely that delayed postconditioning initiated as late as 2 d after focal stroke is effective, for infarction would have been matured 2 d post-stroke. However, it takes at least a few hours for infarction to fully develop, and various studies have demonstrated that drugs, such as recombinant human erythropoietin [11] and huperzine A [12], a reversible and selective acetylcholinesterase (AChE) inhibitor, injected as late as 6 h after stroke, reduce infarct size [11], [12], [13]. Therefore, it is feasible to explore if delayed postconditioning, which is performed a few hours after reperfusion, protects against ischemic brain.

In this study, we tested whether delayed postconditioning, conducted by the repetitive occluding and releasing of the bilateral or ipsilateral common carotid artery (CCA), reduces infarction in focal ischemia in rats; and we further studied the protective effects of delayed postconditioning on metabolism, edema and blood brain barrier (BBB). Moreover, reperfusion for ischemic stroke patients is mainly achieved by tissue plasminogen activator (t-PA) application for dissolving the clot with a limitation of a 3 h therapeutic time window after stroke [14], and its application is complicated increasing hemorrhage and aggravated ischemic injury [15]. Thus, we further tested whether delayed postconditioning eliminates the exaggerating effect of t-PA.

## Materials and Methods

### Focal Cerebral Ischemia

Experimental protocols were approved by the Stanford University Administrative Panel on Laboratory Animal Care (APLAC). Anesthesia was induced by 5% isoflurane and maintained at 1 % to 2 % isoflurane during surgery and early reperfusion in male Sprague–Dawley rats (270 to 330 g). Focal cerebral ischemia was generated by occluding the bilateral CCA for 30 min combined with permanent occlusion of the left distal middle cerebral artery (MCA) above the rhinal fissure, as previously described [2], [4], [16]. Core body temperatures were maintained at 36.5–37.2°C throughout the experiments. The right femoral artery was cannulated for blood collection for the analysis of blood gasses, and arterial pO_2_, pCO_2_ and pH were controlled in normal ranges.

### Infarct size measurement

Acute infarct size was measured as described [3], [17]. Rats were euthanized with an overdose of isoflurane 48 h after stroke, perfused with phosphate buffered saline (PBS), and then the brains were removed and sectioned coronally at 2-mm intervals, generating a total of 5 sections, which were stained with 2% solution of 2,3,4-triphenytetrazolium-chloride (TTC). Using a computerized image analysis system (NIH image, version 1.61), the area of infarction was measured at the sides of the inner section. Infarct size of the ischemic cortex was normalized to the non-ischemic cortex and expressed as a percentage, and an average value from the 5 slices was presented.

For chronic studies, rats were euthanized; brains were removed, post-fixed in 4% paraformaldehyde and 20% sucrose in PBS overnight, frozen, then cut into 30 µm thick coronal sections on a cryostat [16]. These sections were stained with cresyl/violet solution, and the injured cortex was measured and normalized to the contralateral cortex as a percentage, as described [16].

### Postconditioning

To explore optimal parameters for the protective effects of delayed postconditioning, our pilot study tested 60 rats with 41 conditions performed at 0.5h, 1.5h, 3h, 6h after CCA release (Table-1). Postconditioning was executed by occluding either the left or right CCA, or bilateral CCA by using aneurysms clips (Item #, FE 681K, Aesculap Inc, USA); the occlusion time varied from 10 sec, 30 sec, 1 min, 3 min, 5 min, 10 min, 15 min, 30 min to 60 min, and in most cases the CCA or CCAs were released for the same duration and repeated for 3, 5, 6 and 10 cycles; with the exception of one rat the occlusion period was 5 min and 25 min reperfusion, and in a second rat the occlusion period was 5 min and 55 min reperfusion, repeated for 6 cycles, and in a third rat the occlusion period was 15 min and 45 min reperfusion, repeated for 5 cycles. The wound was then covered with wet cotton rinsed with normal saline. In some cases, the ipsilateral CCA or bilateral CCAs were occluded once and then released. Among these, we found that postconditioning performed at 3h and 6h showed a reduction trend in infarction; these conditions include postconditioning with bilateral 30 sec CCA occlusion/30sec release for 3 cycles performed at 3h, and with 15 min left CCA occlusion/15 min release for 6 cycles performed at 3h and 6h. In addition, we have previously studied optimal parameters for rapid postconditioning; we found that postconditioning with 10 cycles of 10 sec reperfusion/10 sec occlusion generated the strongest protection for rapid postconditioning. Thus, it is reasonable to test if this specific cycle for rapid postconditioning still works for delayed postconditioning. Based on these results, we designed the following studies.

**Table 1:** Pilot tests for delayed postconditioning.

Postcon onset time	Vessels invovled	Occlusion time	Reperfusion time	Cycle No.	Rat No.	Infarct size
0h	LCCAO	30 min	To the end*	1	1	34.6
		30 min	30 min	3	1	44.1
	RCCAO	15 min	15 min	3	1	41.0
		5 min	5 min	3	1	30.7
	BCCAO	15 min	15 min	6	1	54.9
1.5 h	LCCAO	30 sec	To the end*	1	1	43.6
		15 min	To the end*	1	1	35.2
		5 min	To the end*	1	1	50.6
3h	BCCAO	10 sec	10 sec	10	5	38.6±11.8
		30 sec	30 sec	3	3	26.8±13.9
		30 sec	30 sec	5	1	43.9
		3 min	3 min	3	1	38.8
		5 min	To the end*	1	2	38.1±6.0
		5 min	5 min	3	1	52.9
		10 min	To the end*	1	1	40.6
		10 min	10 min	3	1	35.5
		15 min	To the end*	1	2	37.7±1.3
		15 min	15 min	3	1	39.8
		30 min	To the end*	1	1	30.2
	LCCAO	15 min	15 min	6	2	23.5±11.9
6 h	BCCAO	10 sec	10 sec	5	1	54.2
		10 sec	10 sec	10	1	37.3
		30 sec	30 sec	3	1	41.5
		30 sec	30 sec	5	1	48.9
		30 sec	30 sec	10	1	52.0
		1 min	1 min	3	1	51.1
		5 min	To the end*	1	7	33.5±14.2
		5 min	5 min	3	1	37.9
		5 min	5 min	6	1	41.5
		10 min	To the end*	1	2	41.56±7.9
		10 min	10 min	2	1	36.2
		15 min	To the end*	1	4	32.29±18.0
		15 min	15 min	2	1	33.12
		15 min	15 min	3	1	43.9
		30 min	To the end*	1	3	40.1±6.7
		60 min	To the end*	1	1	43.09
	LCCAO	15 min	15 min	3	2	29.3±6.4
		15 min	15 min	6	2	15.8±9.0
		5 min	5 min	6	1	42.3

* , for these tests, the occlusion was conducted one time only, so reperfusion was maintained for 2 days till the animals were euthanized. LCCAO, left CCA occlusion; RCCAO, right CCA occlusion; BCCAO, bilateral CCA occlusion.

**Figure 1:**
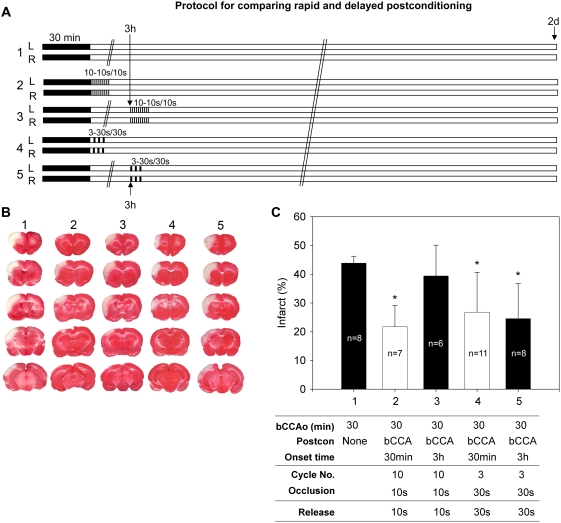
Delayed postconditioning with a series of *bilateral* CCA occlusion and reperfusion reduced infarction. A. The diagram shows the protocols to compare the protective effects of rapid and delayed postconditioning, in which postconditioning was carried out by occluding or releasing the bilateral CCA. Postconditioning was induced by 10 cycles of 10 sec occlusion and 10 sec release of the bilateral CCA (group 2 and 3), or by 3 cycles of 30 sec occlusion and 30 sec release (groups 4 and 5). Rapid postconditioning in groups 2 and 4 was induced immediately after reperfusion; delayed postconditioning in groups 3 and 5 were initiated 3 h after reperfusion. L. left CCA; R, right CCA. B. Representative infarcts stained by TTC from each group. C. Average infarct sizes. Infarct size was measured 2 d after ischemia. Conditions for each group are indicated below the bar. * vs control ischemia (group 1), P<0.05.

First, to detect whether the conditions that generated the strongest protection for rapid postconditioning would reduce infarction at a delayed time window, rats were randomly assigned into 5 groups (Figure 1A): control ischemia without postconditioning; rapid or delayed postconditioning with 10 cycles of bilateral CCA occlusion for 10 sec occlusion/10 sec release, or with 3 cycles of 30 sec occlusion/30 sec release. Rapid postconditioning was performed immediately after reperfusion, and delayed postconditioning was conducted at 3 h after reperfusion.

**Figure 2:**
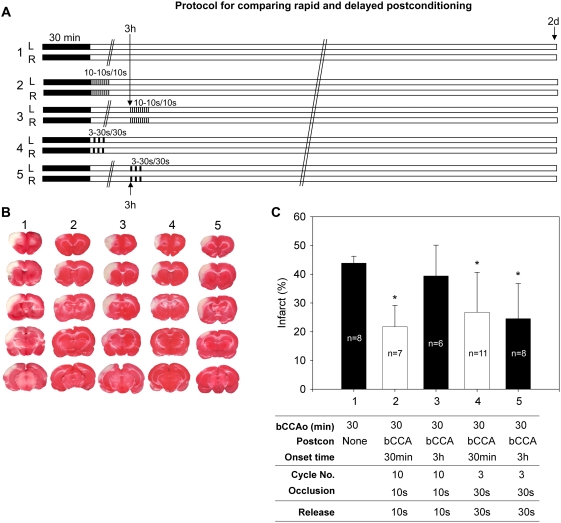
Delayed postconditioning with a series of *ipsilateral* CCA occlusion and reperfusion reduced infarction. A. Comparative timelines for delayed postconditioning, in which postconditioning was conducted by occluding or releasing the ipsilateral CCA alone. Delayed postconditioning was performed by 6 cycles of occluding or releasing the left CCA alone; each occlusion or release lasted for 15 min. Delayed postconditioning was initiated at 3 h (group 2), 6 h (group 3), or 12 h (group 4) after reperfusion. Rats in group 1 received 1–2% isoflurane for 3h starting from 6h after reperfusion; this group serves as a control for postconditioning. L. left CCA; R, right CCA. B. Representative infarcts stained with TTC from each group. C. Average infarct size in rats treated with delayed postconditioning. Conditions for each group are indicated below the bar. *, vs. ischemic control (Group 1), P<0.05; # vs group 2, P<0.05.

Second, to study the effects of delayed postconditioning with manipulation of the ipsilateral CCA, rats were divided into 4 groups (Fig. 2A). While under anesthesia with isoflurane for 3 h, delayed postconditioning was carried out by 6 cycles of occlusion and release of the ipsilateral CCA, starting from 3 h, 6 h and 12 h after reperfusion. Since isoflurane might be neuroprotective, the same period of isoflurane treatment was applied to rats receiving control ischemia only (Fig. 2A). All rats were euthanized 48 h after ischemia for TTC staining and infarct size measurement.

The pilot results showed that delayed postconditioning with 6 cycles of 15 min occlusion/15 min release, which was initiated at 6 h, had the most consistent protective effects. Therefore, this cycle was used for the subsequent experiments, unless specified.

### Definition of ischemic core and penumbra

**Figure 3:**
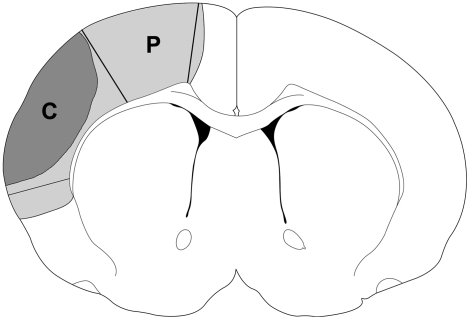
The definition of the ischemic penumbra and core. The gray region (p) plus the black region (C) represent ischemic injury in a control rat with ischemia alone; C represents infarction in a rat that received ischemia plus postconditioning. The region P spared by postconditioning is defined as the penumbra and the region C is defined as the ischemic core.

Ischemic penumbra is arbitrarily defined as the ischemic region saved by ischemic postconditioning 2 days after stroke, while ischemic core refers to the infarct region in rat brains receiving ischemic postconditioning (Fig. 3). For microPET imaging, the ischemic core corresponds to the ischemic region without a clear uptake of 2-[^18^F]-fluoro-2-deoxy-D-glucose (FDG), while the ischemic penumbra defines a region with a relatively weak FDG uptake in the ischemic hemisphere compared to the non-ischemic hemisphere.

### Small animal PET imaging

To detect glucose uptake after stroke with or without delayed postconditioning, microPET study was performed at 11h and 48h after stroke. However, the signaling of glucose uptake 48h after stroke in the ischemic brain was confounded by that of an inflammatory response in the incision on the head, thus, the results from the time point of 48h were not processed. 2-[^18^F]-fluoro-2-deoxy-D-glucose (FDG) PET was performed while the animals were under isoflurane anesthesia (1%–2%) in spontaneous respiration. Two groups were compared: control ischemia without delayed postconditioning and focal ischemia plus delayed postconditioning conducted at 6 h after stroke. ^18^F-FDG (18.5 MBq) was intravenously injected at 10 h after the onset of stroke, and 5 min static PET images were obtained 1 h postinjection. The data was acquired using a microPET R4 rodent model scanner (Siemens Medical Solutions, USA) with a field of view (FOV) centered on the brain region of each rat. The images were reconstructed by a two-dimensional ordered subsets expectation maximum (OSEM) algorithm, with no attenuation or scatter correction. For each microPET scan, three-dimensional regions of interest (ROIs) were drawn over the stroke region and collateral normal brain tissue on decay-corrected coronal images. The average radioactivity concentration within stroke was obtained from mean pixel values and normalized to that of non-ischemic cortex, and expressed as a percentage.

### MR imaging

Delayed postconditioning must be conducted before infarction maturation in order to reduce infarct size. Therefore, we need to confirm that there was no profound infarction before the onset of delayed postconditioning using magnetic resonance imaging (MRI). MR imaging was performed at 5 and 24 h after operation in a GE a 3.0-T whole-body clinical scanner (Systems Revision 12.0 M5; GE Healthcare). Rats were anesthetized during imaging using 1–2% inhaled isoflurane anesthesia. The brain of each rat was imaged with T2-weighted Fast Spin Echo (receiver band width = 16 kHz; repetition time (TR) = 5000 ms; echo time (TE) = 86 ms; echo train length = 8; field of view (FOV) = 4×4 cm; matrix = 256×256; 16 slices; slice thickness = 1 mm; NSA = 3; total imaging time = 8 min). MR images were acquired either coronally—perpendicular to the anterior-posterior (long) axis of the animal, or axially—parallel to the anterior-posterior direction.

### Evaluation of blood-brain barrier integrity

To examine whether delayed postconditioning prevents BBB leakage, BBB integrity was studied using Evans blue [18]. The time course of BBB leakage in rats receiving control ischemia was studied at 6, 24, and 48 h post-stroke onset. Since delayed postconditioning was conducted at 6h, the effect of delayed postconditioning on BBB was examined at 24 h and 48 h after stroke. Evans blue (4%, 2 ml/kg) was injected intravenously (i.v.) into ischemic rats, and the rats were perfused with heparinized saline solution 2 h later. The rat brains were harvested, and the ischemic core and penumbra in the ipsilateral hemisphere and the contralateral cortex were dissected, weighed, homogenized and incubated in 500 µl formamide at 54°C for 2 h. The solution was centrifuged at 12,000 g for 15 minutes; the supernatant was removed and Evans blue was measured using spectrophotometry (absorbance at 620 nm) (Spectro Max 340, Molecular Devices, Sunnyvale, CA, USA). The amount of Evans blue was computed based on external standards in the same solvent (1–20 µg/ml) and expressed as per gram of tissue.

### Edema measurement

To examine the effect of delayed postconditioning on edema, rats were randomly assigned to 3 groups: normal rats without ischemia; ischemic rats without postconditioning; ischemic rats with delayed postconditioning of 6 cycles of 15 min left CCA occlusion/15 min left CCA release. Rats that survived 48 h after stroke were euthanized for edema measurements using the wet-dry weight methods with slight modification [19]. Briefly, after euthanization, the rat brains were sectioned coronally at 2-mm intervals, generating a total of 6 blocks. The ischemic and non-ischemic hemispheres from each block were separated, weighed for wet weight, baked at 90±2°C for 1 week, and weighed again for dry weight. Water content in the brain tissues were calculated as: [1−(dry weight/wet weight)]×100%. The mean values from the left 6 blocks of ischemic hemisphere, and from the right 6 blocks of non-ischemic hemisphere, represent the water content in the ischemic and non-ischemic hemisphere, respectively.

### t-PA treatment

To detect the protective effect of delayed postconditioning on the exaggerative effect of t-PA on brain injury, rats were randomly assigned into 4 groups: control ischemia; ischemia plus t-PA; ischemia plus postconditioning; ischemia plus both t-PA and postconditioning. T-PA (2 mg/kg) was infused intravenously for 1 h starting at 5 h after ischemia; the dosage was adopted from a previous report [20]. Delayed postconditioning with 6 cycles of 15 min left CCA occlusion/15 min release was performed from 6 h after stroke. Rats were euthanized 48h later for TTC staining and infarct size measurement.

### Behavior test

Rats were randomly assigned into 3 groups for behavior tests [16], [21]: rats in the sham surgery group received sham surgery without ischemia and postconditioning; rats in the ischemic group received ischemia and 3 h of isoflurane anesthesia at 6h after stroke without postconditioning; and rats in the postconditioning group received ischemia plus postconditioning with 6 cycles of 15 min left CCA occlusion/15 min release performed at 6 h after stroke onset. We used four standard behavior tests to quantify motor asymmetry caused by a unilateral cortical stroke as described in our previous study. All behavior tests were performed by a person who was blind to the experimental conditions. Most tests were performed before dMCA occlusion and then on days 2, 3, 7, 10, 14, 21, 30, 37, 44 and 51 after dMCA occlusion.

Vibrissae-elicited forelimb placement test, which was used to detect forelimb placing against the edge of a table, was induced by gently brushing the rats' vibrissae on each side; the reflex was tested 10 times on each side per trial, and two trials occurred per test session. The percentage of vibrissa stimulations in which a paw placement occurred was calculated.

For postural reflex test, the rat was placed on a table, and the tail was held by one hand while the other hand gently pushed the animal's shoulder, moving it laterally ∼20 cm. The use of the forelimbs to resist the lateral movement was scored from 0, 1 and 2, with 0 being normal and 2 being no resistance, indicative of severe brain injury as defined in our previous study.

Tail hang test was performed at each time point after stroke as described. Being lifted by the tail, an ischemia-damaged rat will immediately turn to the contralateral (right) side. “Turns” were counted when the angle reached 90° or more. The test was repeated 10 times each testing day. The percentage of trials on which a right turn occurred was calculated.

Home cage limb use test was performed after completing the other behavior tests. The animal was returned to its home cage, and we counted the number of times the rat used its forelimbs to brace itself against the wall; counting separately for the ipsilateral, contralateral, or both forelimbs, until 20 such contacts were reached. The percentage of times out of 20 that the ipsilateral forelimb contacted was computed using this formula: (ipsilateral+(both/2))/20×100%.

### Statistical Analyses

All data was collected and analyzed in a blind fashion. Statistics were performed using the 3.1 version of SigmaStat (Systat Software, Inc, California, USA). Since the animal number of each group is mostly below 10, non-parametric statistic tests were used to compare infarct sizes, edema and BBB leakage (Mann-Whitney rank sum test was used to compare the difference between two groups; when there are more than two groups to compare, Kruskal-Wallis ANOVA on Ranks was used, followed by Dunn's Multiple Comparison Tests). For microPET image, Kruskal-Wallis ANOVA on Ranks was used to compare between slices within a group; two-way anova was used to compare between postconditioning and control ischemia, followed by Student–Newman–Keuls test. For behavioral tests, the Friedman Repeated Measures ANOVA on Ranks was used to compare tests at different time points in the same group and Two Way Repeated Measures ANOVA was used to compare between groups, followed by Student–Newman–Keuls test. Tests were considered statistically significant at P-values <0.05. Data are presented as means±sd.

## Results

### Delayed postconditioning reduced infarction

We have previously found that rapid postconditioning with10 cycles of 10 sec release/10 sec occlusion of the bCCA, provided the strongest protection among various parameters for rapid postconditioning [3]; however, it did not reduce infarction when performed at 3 h after stroke (group 3) (Fig. 1). Nevertheless, with 3 cycles of 30 sec release/30 sec occlusion of the bCCA, both rapid postconditioning conducted at reperfusion (group 4) and delayed postconditioning at 3 h (group 5) mitigated infarction by 39% and 44%, respectively, compared with control ischemia (group 1) (Fig. 1).

We then tested the effects of delayed postconditioning with 6 cycles of 15 min occlusion/15 min release of the ipsilateral CCA. When executed at 3h and 6h after stroke, delayed postconditioning reduced infarct size by 46% and 64%, respectively (Fig. 2). It appears that the latter cycle offered the strongest protection; however, such protective effect was lost when performed at 12h after stroke.

**Figure 4:**
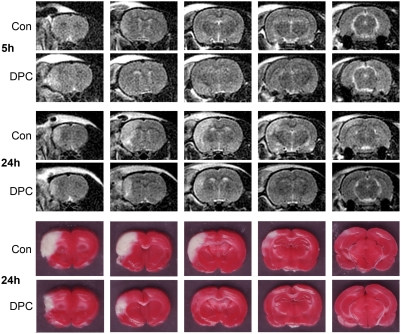
T2-weighted magnetic resonance imaging (MRI) confirmed infarction evolution after stroke and delayed postconditioning. Representative MRI images at 5 h (top two rows) and 24 h (middle two rows) from rats subjected to control ischemia and delayed postconditioning after stroke are shown. Control ischemia (con) was induced by 30 min bCCA occlusion combined with permanent dMCA occlusion; delayed postconditioning (DPC) was conducted from 6 h to 9 h by 6 cycles of 15 min release/15 min occlusion of the ipsilateral CCA. TTC staining showed infarction (bottom two rows) corroborated T2 weighted MRI. The rats were euthanized after MRI. Rat brains were sliced into 5 coronal slices and stained with TTC. The infarct region revealed by TTC staining is consistent with that revealed by MRI.

T2-Weighted MRI further confirmed that infarction had matured at 24 h in the cortex after control ischemia, which was prevented by delayed postconditioning (Fig. 4); however, there was no obvious differences at 5 h between the two groups.

### Delayed postconditioning offered long-term protection and preserved neurological function

We next detected the chronicle protective effect of delayed postconditioning with 6 cycles of 15 min occlusion/15 min release of the ipsilateral CCA performed at 6 h on behavior tests.

**Figure 5:**
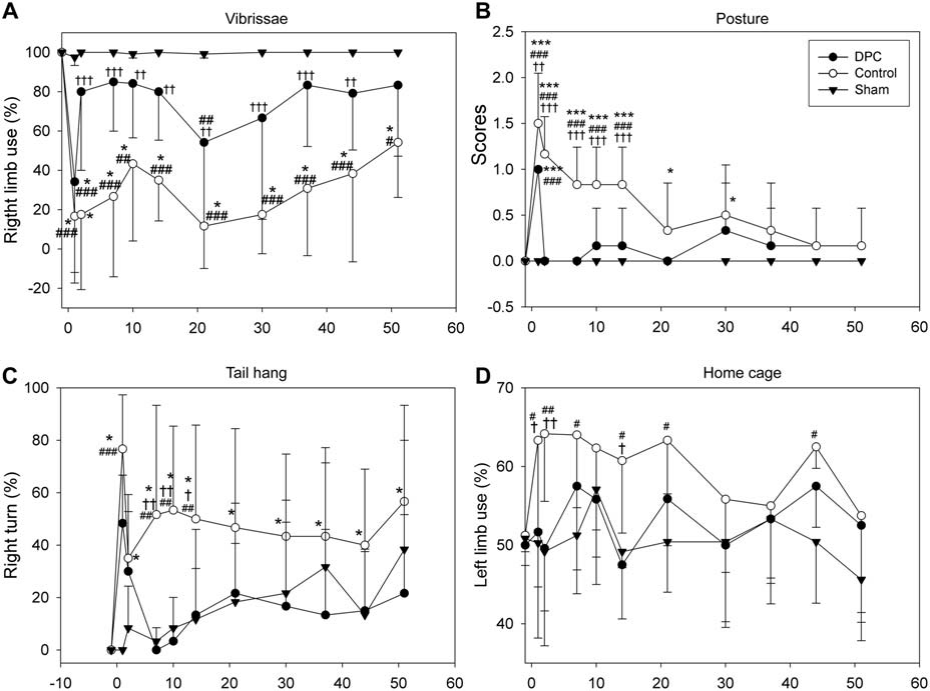
Delayed postconditioning attenuated behavioral deficits up to 2 months post-ischemia. Delayed postconditioning (DPC) was conducted with 6 cycles of 15 min occlusion/15 min release in the ipsilateral CCA occlusion 6 h after stroke. Four tests were performed: A. Vibrissae-elicited forelimb placement test. All sham rats showed normal forelimb placing. There was unsuccessful placing of the contralateral forelimb after stroke in control rats; postconditioning attenuated the overall deficit from 1 to 51 d after stroke. The analysis of Two-way repeated measures ANOVA show that there are significant differences between groups: sham vs DPC, P = 0.037; sham vs control ischemia, P<0.001; DPC vs control ischemia, P = 0.003. * vs. before ischemia, P<0.5; #, ##, ###vs. sham, P<0.05, 0.01, 0.001; ††, †††, vs. postconditioning, P<0.01, 0.001. N = 6/each group. B. Postural reflex test. Scores significantly increased in control rats at 1, 2, 7, 10, and 21 d after stroke; postconditioning reduced scores at 1, 2, 7, 10 d after stroke. Two-way repeated measures ANOVA shows that there are significant differences between groups: sham vs DPC, P = 0.058; sham vs control ischemia, P<0.001; DPC vs control ischemia, P<0.001.. *, *** vs. before ischemia, P<0.01, 0.001. ### vs. sham, P<0.001; ††,††† vs. postcon, P<0.01, 0.001; . C.Tail hang test. The number of large right turns induced by tail hanging increased in control rats; postconditioning blocked right turns at 7, 10, and 14 d. Two-way repeated measures ANOVA: Control vs sham, P = 0.022; control vs DPC, P = 0.014; DPC vs sham, P = 0.805. *, vs. before ischemia, P<0.05; ##, ###, vs. sham, P<0.01, 0.001; †, ††, vs. postconditioning, P<0.05, 0.01. D. Home cage forelimb use test. Left-limb-use increased at 1, 2, 7, 10, 14, 21 and 44 d, which was blocked by postconditioning. Two-way repeated measures ANOVA: control ischemia vs sham, P = 0.004; control ischemia vs DPC, P = 0.010; DPC vs sham, P = 0.375. # , ## vs. sham, P<0.05, 0.001; †, ††, vs. postcon, P<0.05, 0.001; ** vs. before ischemia.

Four standard behavioral tests were performed to evaluate delayed postconditioning's protection on neurological function (Fig. 5). In the vibrissae test, placing of the forelimb contralateral to the injury was disrupted from 1 to 51 d after stroke in control ischemic rats; delayed postconditioning did not prevent such disruption at 1d but attenuated it thereafter (Fig. 5A). The scores for the postural reflex test increased at 1 and 2 d, and remained high until 14 d, then gradually decreased to normal levels in control rats; the scores were attenuated by delayed postconditioning from 1 to 14 d (Fig. 5B). Tail hang test showed that delayed postconditioning significantly attenuated the percentage of large right turns in control ischemic rats (Fig. 5C). Lastly, in the home cage test, control ischemic rats showed a bias in favor of the ipsilateral forelimb in most days from 1 to 44 d after stroke; delayed postconditioning abolished this bias (Fig. 5D).

The histological results demonstrated that delayed postconditioning reduced ischemic injury size by 40 % at 2 months after stroke (36.7+/−14% for control ischemia and 22.4+/−19.7% for delayed postconditioning, no significant difference between the two groups, P = 0.185)

### Delayed postconditioning improved glucose uptake

**Figure 6:**
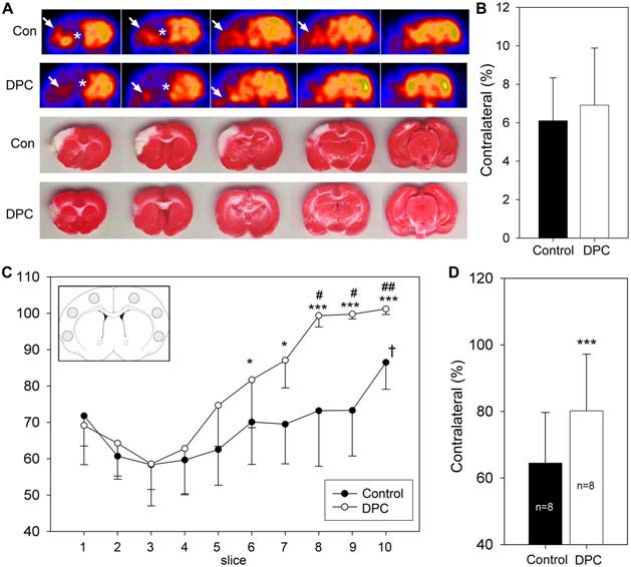
Effects of delayed postconditioning on FDG uptake. A. Representative PET imaging for FDG uptake and corresponding TTC staining. PET imaging was conducted 11 h after stroke; delayed postconditioning (DPC) was 6 cycles of 15 min occlusion/15 min release in the ipsilateral CCA. In the ischemic core, no apparent FDG uptake was detected (* indicated by the dark region defined as the ischemic core); in the penumbra, the signal was weaker than that in the contralateral hemisphere. There is a non-specific signal caused by the skin incision outside the brain (arrow). Rats were euthanized 2 d after stroke for TTC staining. The infarct region corroborates with the region with reduced FDG uptake. B. Delayed postconditioning (DPC) did not affect ischemic core sizes measured from PET imaging. PET images from a total of 10 levels, with a 1 mm distance between adjacent levels, were selected. The core area was measured and normalized to the whole contralateral hemisphere and expressed as a percentage; an average value from all measured levels is presented. There was no difference in the core size between rats receiving control ischemia and delayed postconditioning, suggesting that delayed postconditioning did not change FDG uptake in the ischemic core. C. DPC improved FDG uptake. The optical densities of Region of interest (ROI) in the selected 10 coronal slices from rostral (level 1) to caudal (level 10) were measured; ROIs are indicated on the inserted diagram (right corner). Optical densities in three ROIs (1 mm diameter circle) in the cortex from the ipsilateral and contraleral cortex were measured. The average density in the ipsilateral hemisphere was divided by that in the contralateral hemisphere and expressed as a percentage. FDG uptake is significantly higher in the caudal slices (slices 5–10) vs. rostral slices (slices 1–4) in rats receiving DPC; it is also significantly higher in slice 10 than in other slices in rats receiving control ischemia. DPC improved FDG uptake in slices 5 to 10 compared with corresponding slices in control rats. *, *** vs. corresponding time points in control rats, P<0.05, 0.001, respectively. † vs slice 2,3,4,5, P<0.05;. # vs. slice 1,2,3,4, P<0.01; ##, vs. slice 1, 2, 3, 4, 5, P<0.05 (Anova on Ranks followed with Dunn's test). *, *** P<0.05, 0.001, vs corresponding slices in the DPC groups. D. Square means of overall values from all 10 slices. DPC significantly improved overall FDG uptake. *** vs. control ischemia, P<0.001.

Metabolisms reflected by FDG-uptake was measured 11 h after stroke by PET imaging (Fig. 6). FDG-uptake was reduced in the ischemic hemisphere; the reduced region corresponds to the infarction stained by TTC (Fig. 6A). All ischemic brains, with or without delayed postconditioning, showed an apparent ischemic core where FDG uptake was near zero, as reflected by the dark region on the PET imaging. The size of this dark region, the ischemic core, was measured. The core size did not differ between animals with and without delayed postconditioning (Fig. 6B). However, delayed postconditioning robustly improved the overall FDG (Fig. 6 C,D). We also confirmed that delayed postconditioning reduced infarct size in rats used in the microPET study (data not shown).

### Delayed postconditioning attenuated stroke-induced edema

**Figure 7:**
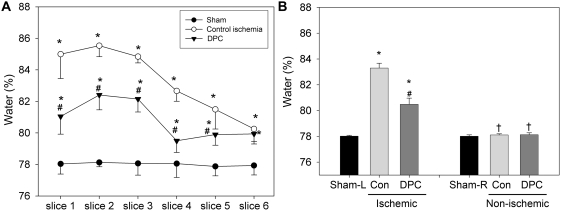
Delayed postconditioning (DPC) mitigated edema after stroke. Rat brains were harvested at 48 h after stroke for edema measurement. The rat brains were sectioned coronally at 2 mm intervals, generating a total of 6 blocks from rostral to caudal. A. Rostral to caudal slice water content. Water contents in all 6 slices are presented. * vs. sham, P<0.001; # vs. control, P<0.001. B. Mean water content from all slices of the ischemic and non-ischemic hemisphere from rats with sham surgery, control ischemia, and delayed postconditioning. The mean value from the 6 blocks of each hemisphere was calculated, and a mean value from all rats (n = 6/group) is presented. DPC attenuated overall edema after stroke. Ischemic, ischemic hemisphere; non-ischemic, non-ischemic hemisphere; con, control ischemia. * vs. Sham-L, P<0.05; # vs. con, P<0.05; † vs. ischemic hemisphere, P<0.05.

Brain edema was detected 2 d after stroke by the wet-dry method [19]. The rostral slices contained more water than the caudal slices, and more water content was detected in rats receiving stroke with and without postconditioning than in sham rats without ischemia. However, delayed postconditioning significantly reduced water content from slices 1 to 5 compared with those in control ischemic rats. In general, water content was increased from ∼78% in the non-ischemic brain to ∼83% in the ischemic hemisphere 2 d after stroke (Fig. 7). Although water content was increased to ∼80% in the ischemic brain with delayed postconditioning, it is significantly lower than that of control ischemic brain (Fig. 7), suggesting that delayed postconditioning attenuated stroke-induced edema.

### Delayed postconditioning reduced BBB leakage

**Figure 8:**
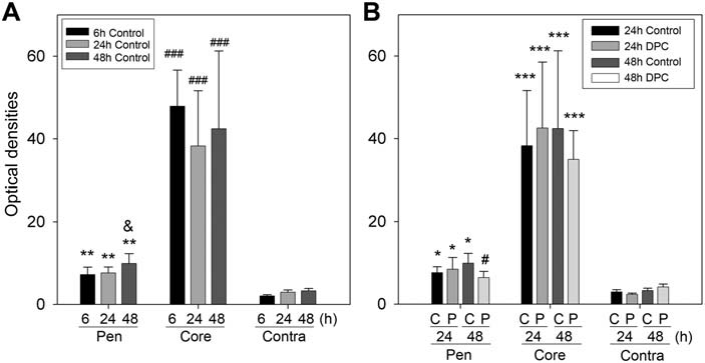
Delayed postconditioning (DPC) inhibited BBB leakage. The ischemic core and penumbra and the non-ischemic hemisphere were dissected for Evan's blue detection. A. Time course of BBB leakage after stroke. The amount of Evan's blue in the ischemic and non-ischemic hemisphere was detected at 6, 24, and 48 h after stroke. Evan's blue penetrated into the ischemic brain tissue as early as 6 h, and persisted up to 48 h. More leakage occurred in the core than in the penumbra. Pen, penumbra; contra, contralateral hemisphere. ** vs. corresponding contralateral hemisphere, P<0.01; ### vs. corresponding penumbra and contralateral hemisphere, P<0.001; & vs. 6 h and 24 h in the penumbra, P<0.05 (Rank sum test). B. The effect of DPC on BBB was detected at 24 h and 48 h after stroke. DPC reduced BBB leakage at 48 h but not at 24 h after stroke in the penumbra; it had no effect on BBB leakage in the ischemic core. N = 5–6/group. C, control ischemia; P, delayed postconditioning. * vs. corresponding contralateral hemisphere, P<0.05; *** vs. corresponding contralateral hemisphere or penumbra, P<0.001. # vs. 48 h-control penumbra, P<0.05.

BBB permeability was measured by Evan's blue. The content of Evan's blue increased as early as 6h, and remained unchanged until 6h, but robustly increased at 48 h after stroke in the ischemic penumbra (Fig. 8), suggesting a time-dependent BBB leakage. However, BBB leakage reached a peak at 6 h in the ischemic core, and further increases in BBB leakage were not detected at 24 h and 48 h. Much higher content of Evan's blue was detected in the core than in the penumbra. Delayed postconditioning blocked BBB leakage at 48h but had no effect on core's BBB leakage at 24h in the penumbra.

### t-PA exacerbated-brain injury was inhibited by delayed postconditioning

**Figure 9:**
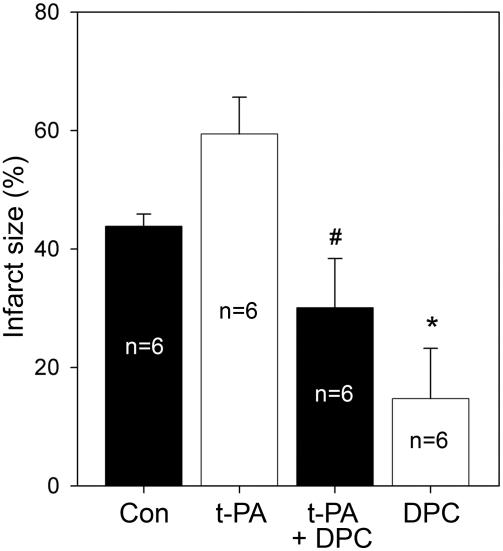
Delayed postconditioning (DPC) attenuated t-PA's worsening effect on infarction. T-PA (2 mg/kg) was injected intravenously for 1h started at 5 h after ischemia of 30 min bCCA occlusion plus permanent dMCA occlusion; postconditioning with 6 cycles of 15 min occlusion/15 release of the left CCA was performed from 6 h after stroke. Rats were killed 48h later for TTC staining and infarct size measurement. T-PA injection significantly worsened ischemic injury compared with control ischemia; delayed postconditioning attenuated its exacerbating effect. N = 6/group. * P<0.05 vs con or t-PA; # P<0.05,vs t-PA (ANOVA on ranks).

The infarct size in rat brain treated with t-PA was larger than that in control ischemic brain (Fig. 9), which is consistent with previous studies. Delayed postconditioning reduced infarction compared with control ischemia and ischemia plus t-PA (Fig. 9).

## Discussion

For the first time we have demonstrated that delayed postconditioning performed a few hours after reperfusion of focal ischemia provided long-term protection. The protective effect of delayed postconditioning could be achieved by occluding the ipsilateral CCA, which is clinically relevant, for the ipsilateral CCA is accessible. In addition, delayed postconditioning also improved glucose uptake, inhibited edema and mitigated BBB leakage in the penumbra, and lastly, attenuated the exacerbating effect of t-PA.

We found that the optimal parameters for delayed postconditioning, performed a few hours later, differ from that for rapid postconditioning. We and others have previously reported that the protective effects of rapid postconditioning depend on the degree of ischemia [2], the onset time of postconditioning [3], [7], and the cycle number of occluding and releasing the blood vessels [3]. Among these parameters, we found that rapid postconditioning with 10 cycles of 10 sec occlusion/10 sec release of the bilateral CCA offers the strongest protection [3]. However, delayed postconditioning with 10 cycles of 10 sec occlusion/10 sec release performed at 3h did not offer protection, while 3 cycles of 30 sec/30 sec performed at the same time after stroke markedly attenuated brain injury.

We further found that the protective effect of delayed postconditioning can be achieved by 6 cycles of occlusion and release of the ipsilateral CCA, initiated as late as 6h post-stroke. MR imaging confirmed that delayed postconditioning might block infarction evolution, as infarction was developed at 24 h in the ischemic rat receiving sham postconditioning, whereas there was no clear difference in the imaging at 5 h post-stroke between both animals. Therefore, this experiment implies that delayed postconditioning was initiated at a time point when the ischemic brain tissue might still be alive. Nevertheless, there is a limitation in our study that T2 weighted sequence does not exclude infarct development post stroke. A further study using diffusion weighted imaging (DWI) is needed to clarify this issue.

The delayed postconditioning conducted using the ipsilateral CCA may have some advantages over that using the bilateral CCA, as we have shown that the bilateral CCA occlusion causes severe, though short, reduction in CBF[22]; postconditioning with such additional repetitive ischemia after a major stroke, if not well controlled, may further endanger the ischemic brain. Nevertheless, postconditioning conducted by occluding the ipsilateral CCA to the ischemic hemisphere only mildly reduced CBF during each occlusion (data not shown), thus minimizing the potential endangering effect of occluding the bilateral CCA during postconditioning.

The ischemic model and the method of performing postconditioning in our study are clinically important. First, the ischemic model generally mimics frequent clinical cases in which partial reperfusion occur. Most spontaneous recanalization after stroke results in partial reperfusion [23], and further, t-PA treatment leads to partial reperfusion in most stroke patients [24]. In addition, patients with carotid artery stenosis after stroke often receive carotid artery endarterectomy, or angioplasty and stenting for revascularization [25]. If high grade stenosis of the carotid artery is resolved after stroke, partial reperfusion will occur in the ischemic region. In our study, ischemia is generated by bilateral CCA occlusion combined with distal MCA occlusion, as described originally by Chen and colleagues [26]. Two CCAs were released after 30 min of occlusion while the distal MCA remained occluded; the CCA release allowed partial reperfusion and reperfusion was not detected in the ischemic core after CCA release. Therefore, the model used in our study generally mimics partial reperfusion after stroke that frequently occurs in patients; moreover, the damaged region of the cortex is the one most likely detected in human stroke patients. Second, our ischemic model is highly reproducible and reliable [2], [16], [26], [27], [28]. The reliability of an ischemic model is essential to characterize the efficacy as well as the protective mechanisms of a neuroprotectant such as postconditioning. Third, and most importantly, the methods used to generate delayed postconditioning in our study are highly clinically relevant, since the cervical carotid arteries are accessible. In fact, physicians often occlude the carotid artery during the insertion of guiding catheters [29], or for temporary balloon occlusion for the treatment of intracranial aneurysms and tumors [30]. Carotid endarterectomy is actually widely used for stroke prevention; therefore, briefly occluding the carotid artery has been proven safe [31].

The clinical importance of our study is further strengthened by the fact that delayed postconditioning attenuated the worsening effect of t-PA. For ischemic stroke patients, reperfusion is mainly achieved by t-PA application, which is the only FDA approved pharmacological agent that dissolves blood clots for acute stroke treatment [14]. However, the use of t-PA is limited by a 3 h therapeutic time window after stroke, and is complicated by its side effects of increasing symptomatic intracerebral hemorrhage [14]. It has been well-established in the laboratory that t-PA is neurotoxic, and its application increases ischemic injury under certain conditions [32]. Thus, t-PA therapy combined with neuroprotectants has been explored aiming at reducing its neurotoxicity [15]. In our current study, we found that t-PA treatment increased infarct size, but such devastating effect was attenuated by delayed postconditioning, suggesting that delayed postconditioning may be applicable in combination with t-PA treatment for ischemic stroke patients.

The protection of delayed postconditioning may be achieved by its ability to improve metabolism after stroke. Compelling evidence in this study showed that delayed postconditioning with ipsilateral CCA occlusion improved FDG uptake as detected by PET imaging. However, it is unknown why delayed postconditioning is able to improve metabolism; nevertheless, postconditioning is performed directly on blood vessels, and it has been reported to improve endothelial function [33], [34]. In addition, we have previously shown that rapid postconditioning improves CBF recovery after reperfusion [22]. Therefore, delayed postconditioning may also improve metabolisms by improving endothelial function and CBF.

The inhibiting effect of delayed postconditioning on BBB leakage may not play critical roles for its neuroprotection. Although delayed postconditioning mitigated BBB permeability at 48h, it had no effect at 24h, and BBB was open as early as 6h before the induction of postconditioning. Therefore, inhibition of BBB leakage at 48 h by delayed postconditioning may merely reflect the result of postconditioning's protection, rather than the cause by which postconditioning reduces infarction.

The molecular mechanisms of delayed postconditioning are unknown. We have shown that rapid postconditioning blunts production of reactive oxygen species or free radicals, and it inhibits apoptosis in the penumbra [2]. More recently, we and others have shown that Akt activity contributes to the protection offered by rapid postconditioning [4], [7]. The protective mechanisms of rapid postconditioning are also associated with the MAPK and PKC pathways [4], [7]. Whether delayed postconditioning has protective mechanisms in common with rapid postconditioning needs further study.

Whether isoflurane protects against cerebral ischemia remains controversial [35]. Some studies have demonstrated that isoflurane applied during ischemia protects the ischemic brain; its protective effects dependent on the severity of the ischemia [35]. In addition, isoflurane preconditioning [36] and isoflurane postconditioning [37] have also been shown to reduce ischemic injury. However, many reports disagree, which have shown that isoflurane provides little protection or even worsens ischemic damage[38], [39], [40], [41]. We did not find any protection of pre-treatment with isoflurane, either [17]. Even though isoflurane was used to anesthetize the rats in our current study, it is essential to make sure that the protective effect of delayed postconditioning was not caused by isoflurane. Thus, the rats subjected to control ischemia without delayed postconditioning were treated with 1.5% isoflurane for the same period of 3 h that was received by rats with delayed postconditioning. Therefore, reduction in infarct size in our study was due to the delayed postconditioning rather than the isoflurane.

In conclusion, delayed postconditioning protected against focal ischemia in rats. It appeared to provide long-term protection and improved neurological function, and partially reversed the detrimental effect of t-PA. This novel protective model offers an alternative avenue for studying therapeutic strategies against stroke.
