# Correction: Delayed Postconditioning Protects against Focal Ischemic Brain Injury in Rats

**DOI:** 10.1371/annotation/bbfdac40-32cc-4c3a-a049-436796875bf4

**Published:** 2009-02-19

**Authors:** Chuancheng Ren, Xuwen Gao, Gang Niu, Zhimin Yan, Xiaoyuan Chen, Heng Zhao

Figures 1 and 2 do not appear correctly. Figure 2 should be considered Figure 1, and the correct file for Figure 2 can be viewed here: 

**Figure:**
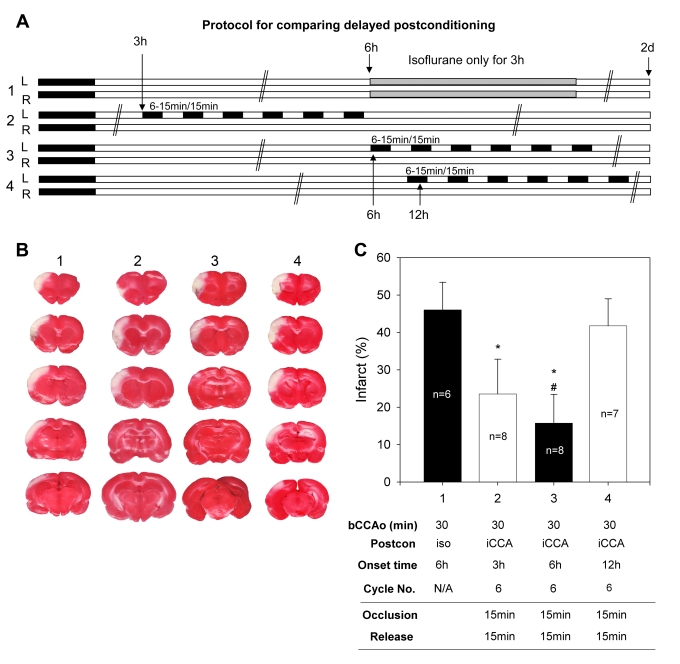


The figure titles and legends are not affected and are correct as they appear.

